# Ending HIV Transmission in Australia: Expanding PrEP to Cisgender Women: A Scoping Review

**DOI:** 10.1007/s10461-024-04386-z

**Published:** 2024-05-28

**Authors:** Catherine MacPhail, Kate Manlik, Hannah Dews, Limin Mao, Alison Rutherford

**Affiliations:** 1 School of Health and Society, University of Wollongong, Wollongong, Australia; 2 Centre for Social Research in Health, University of New South Wales, Sydney, Australia; 3 Illawarra Shoalhaven Local Health District, Wollongong, NSW Australia

**Keywords:** HIV, Prevention, Cisgender Women, Pre-exposure Prophylaxis, PrEP

## Abstract

Pre-exposure prophylaxis (PrEP) availability through the Pharmaceutical Benefits Scheme provides real potential for the elimination of HIV transmission in Australia, as evidenced by a rapid decline in HIV incidence among gay and bisexual men (GBM). However, HIV elimination will not be possible without also extending PrEP to other populations, including cisgender women. We conducted a scoping review to examine the extent to which PrEP access for cisgender women has been considered in Australia. A comprehensive search across five databases, grey literature, and hand search of references was conducted. A single reviewer conducted title and abstract screening and two reviewers completed full-text screening and data extraction. Nineteen documents were included in the final review and included both peer-reviewed journal articles and guidelines and strategies. Focused discussion of cisgender women’s use of PrEP was largely missing from the literature and, although their use of PrEP is supported in some relevant guidelines, little has been done to actively develop strategies to inform cisgender women about PrEP as a precursor to prescribing for HIV prevention. Healthcare providers’ narrow view of PrEP as being the domain of GBM further limits cisgender women’s potential access. If HIV elimination in Australia is to be a reality, we need to develop mechanisms to specifically engage with cisgender women about PrEP.

## Introduction

The UN Sustainable Development Goals, 2018–2022 Australian National HIV Strategy and Agenda 2025 consensus statement target the elimination of new HIV acquisitions in Australia [1–3]. Progress towards this goal has been facilitated by a range of strategies, including alliance between community, government, and health care providers [4]; risk reduction practices such as condom use and serosorting among gay men [5] and advocacy for needle and syringe exchange among people who inject drugs [6]; effective use of Prevention of Mother to Child Transmission (PMTCT) [7]; and the introduction of pre-exposure prophylaxis (PrEP). The availability of PrEP led to a 25% drop in in HIV acquisitions among gay, bisexual and men who have sex with men (GBM) enrolled in the EPIC-NSW study as PrEP transitioned to the Pharmaceutical Benefits Scheme (PBS) in 2018 [8]. Promotion and uptake of PrEP has focused specifically on GBM as the populations most at-risk of HIV acquisition in Australia. There are, however, other populations for whom PrEP may offer an effective strategy to limit HIV risk [9, 10], and to whom very little attention has been paid. Cisgender women are one such population and will require additional focus if Australia is to eliminate the transmission of HIV [1, 2].

**Fig. 1:**
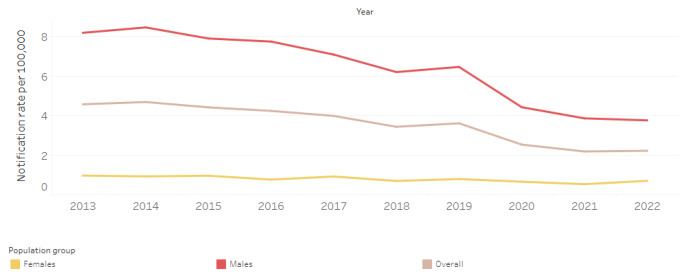
HIV notification rate per 100,000 population [59]

Just under 30,000 individuals were estimated to be living with HIV in Australia in 2022 (0.14% of all Australian adults). Women account for approximately 12% or 3,680 individuals living with HIV [11]. Although GBM remain the highest at-risk population, roughly 100 women in Australia are newly diagnosed with HIV annually [12]. There have been significant declines in new HIV acquisitions in Australia since 2014, but these have been concentrated among GBM [13]. Similar success has not been achieved with other populations and, indeed, women’s HIV incidence has remained relatively unchanged over time [14]. See Fig. 1 from The Kirby Institute’s HIV surveillance data dashboard.

Several sub-populations of cisgender women have been identified as being at heightened risk of acquiring HIV, so would particularly benefit from increased awareness of and access to PrEP. These include Aboriginal and Torres Strait Islander women; women from multicultural backgrounds; and women in relationships with HIV-positive men who struggle to achieve viral suppression, are newly diagnosed, or have recently initiated antiretroviral treatment [15]. Additionally, women who plan to have sex when travelling to high HIV prevalence countries and women working outside of Australia in high endemic countries may be potential PrEP users [16].

### Use of PrEP for HIV Prevention among Cisgender Women in Low Endemicity Countries

Little is known about cisgender women’s need for PrEP, knowledge of its availability, or access outside of high-prevalence low-income countries [9]. A few previous reviews of PrEP use among cisgender women have been undertaken, however none are specifically relevant for cisgender women in Australia. O’Malley et al. [17] have reviewed interventions that target the intersection of PrEP and intimate partner violence. The studies included in their review were all clinical trials in African countries or the US where PrEP availability is very different to Australia. Zhang et al. [18] reviewed the role of PrEP among women who use drugs in the US, specifically noting that PrEP is underutilised by this population. African American and Hispanic women dominated samples in the included studies. Mwaturura et al. [19] have reviewed PrEP use among African migrants in a range of high-income countries (including a single study from Australia). When considering cisgender women more generally, reviews have concentrated on the US experience [20, 21], and therefore focused on US-specific issues such as increased risk associated with distinct minority ethnic backgrounds (African American, Hispanic and Latina women) and the cost challenge of healthcare access, both of which have limited relevance in the Australian context. Baldwin et al. [20] and others have noted that there is a large unmet need for PrEP among cisgender women in the US, with Cernasev et al. [22] explaining that “it is imperative to make women’s voices heard through conducting more research, ensuring sufficient access to PrEP, and enhancing knowledge about PrEP as a viable prevention strategy for women” (p. 123).

Given that mainstream information sources in Australia have been largely silent on PrEP for cisgender women, this paper reports on a scoping review that was conducted to specifically examine whether and how they have been included in discourse around PrEP in Australia and to systematically map resources in this area. The scoping review was guided by the question: What is known from research literature and other documents about the role of PrEP for HIV prevention among cisgender Australian women?

## Materials and methods

This scoping review followed the Preferred Reporting Items for Systematic Reviews and Meta-Analyses (PRISMA ScR) guidelines [23]. The protocol was registered with the Open Science Network at https://osf.io/jfzsx/ .

### Search Strategy

We developed a search strategy that aimed to locate records that discussed cisgender women’s access to and use of PrEP in the Australian environment. Table 1 shows the main search terms and synonyms used. We searched for literature between 2000 and 2023, as PrEP was first approved for use in HIV prevention in 2012 and placed on the PBS in 2018. This date range therefore includes potential research prior to availability. Databases searched included PubMed, Web of Science, CINHAL, SCOPUS and PsychInfo. We also conducted a search in Google for grey literature that we would have missed from database searches. The search was conducted on an incognito browser and included the same search terms as indicated in Table 1, with the addition of the search being directed to intext. We planned to include the first 100 documents of the Google search, but only 95 were generated and were screened and assessed for eligibility. The reference lists of documents included in the review were also screened to identify additional records of interest to the review.

**Table 1:** Search strategy

Major Search Term	Synonyms
PrEP	(PrEP OR “pre-exposure prophylaxis” OR “preexposure prophylaxis” OR combination OR tenofovir OR truvada OR racivir OR emtricitabine)
HIV	AND (HIV OR “Human Immunodeficiency virus”)
Women	AND (wom? n OR female)
Australia	AND (NSW OR “New South Wales” OR Victoria OR Tasmania OR Queensland OR “Northern Territory” OR ACT OR Australia*)

### Inclusion and Exclusion Criteria

Documents that discussed PrEP for cisgender women in Australia were included. Peer-reviewed articles published in English based on all study designs (qualitative, quantitative and mixed method) were included in the review. Included documents had to include cisgender women as a target population for PrEP use – either as the exclusive focus or in a manner that disaggregated them from other populations also discussed. We included documents such as strategies, guidelines, and policies if they specifically discussed PrEP for cisgender women. Documents were excluded if they: (1) focused only on PrEP use for HIV prevention among GBM or trans-women; (2) focused on PrEP for HIV prevention among cisgender women outside of Australia; (3) were other systematic reviews (in order to avoid double reporting of results); (4) focused on HIV treatment; (5) were laboratory studies; (6) general news items about availability of PrEP; or (7) had a focus on microbicides for HIV prevention. We specifically excluded documents focused on microbicides as there are no microbicides currently licenced for use [24]. In most instances, documents focused on microbicide-use were otherwise ineligible for inclusion because they were laboratory-focused or not Australian.

### Data Extraction and Synthesis

**Fig. 2:**
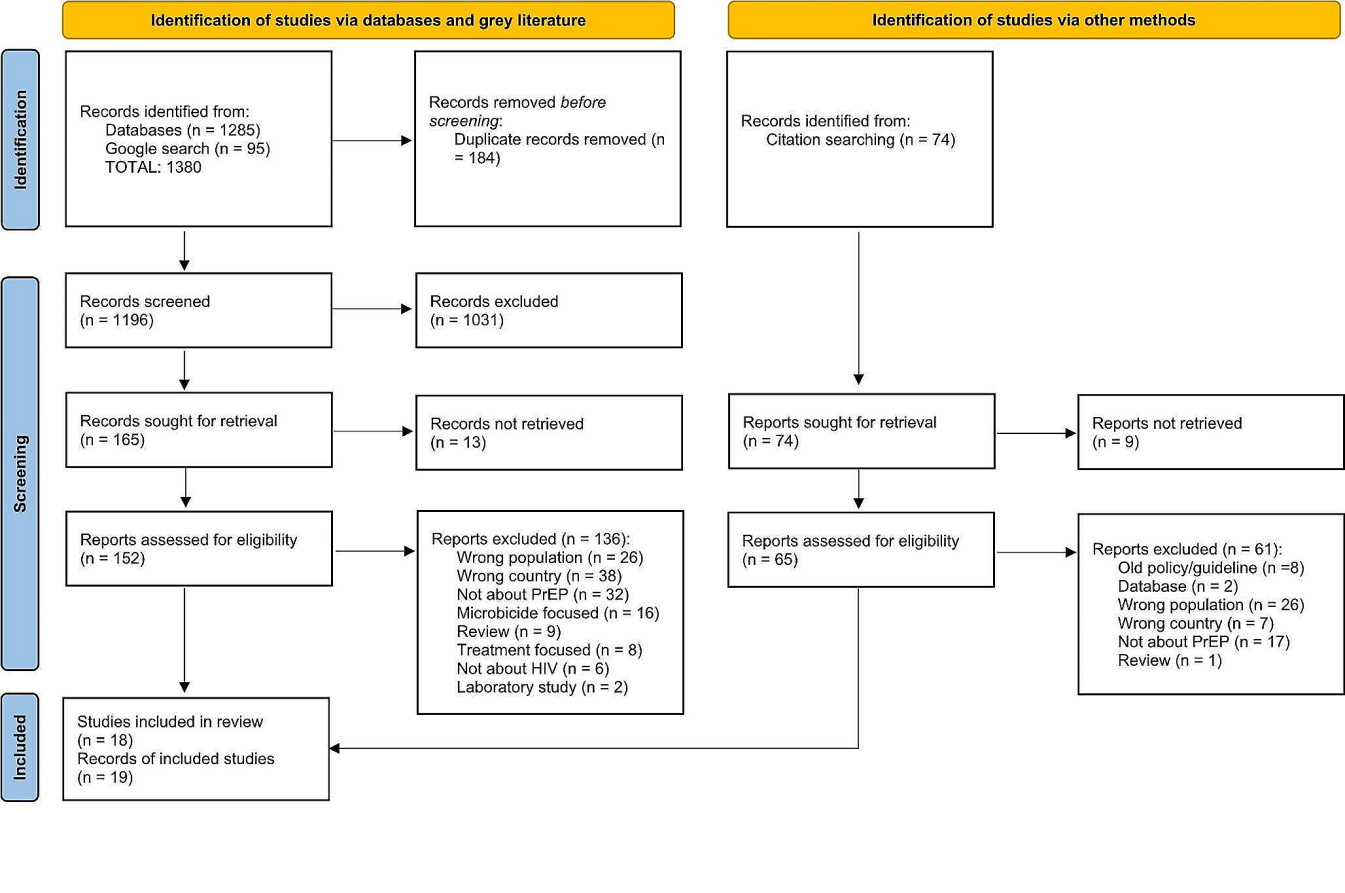
Screening processes undertaken according to PRISMA-ScR guidelines

All records retrieved during the search process were exported and duplicates removed. Titles and abstracts were screened by a single author (either CM or HD) and those that did not meet the eligibility criteria were excluded. The remaining records were retrieved in full and assessed for inclusion using the inclusion and exclusion criteria. Full text review was conducted by two authors (CM & KM or HD) using Covidence.org with the authors blind to one another’s decisions. Where there were differences in the decision to exclude or include articles, the two authors discussed and resolved the decision together. Where there was agreement about exclusion, but the reason was different, a hierarchy of reasons was established, and the first on the hierarchy recorded as the final decision reason. The PRISMA flow diagram (Fig. 2) provides the reasons for exclusion.

Data extraction was conducted using two separate templates developed for this study. The first was used specifically for peer-reviewed articles and the second for documents that were strategies or guidelines. Items included in the data extraction table for peer-reviewed articles included author, year, location, study aim, population, inclusion and exclusion criteria, method of recruitment, number of participants, main study outcome, additional outcomes specifically relevant to cisgender women, focus issues, and study limitations. For strategy and guideline documents, the extraction template included author, year, type of document, aim of document, populations included, main discussion points, discussion points specific to cisgender women, and focus issues. The extraction was undertaken independently by two authors (CM & KM) and the final extraction tables compared and discussed for finalisation.

In synthesising the review findings, we grouped documents as either published research outcomes or as guidelines and strategies. As well as specifically examining the documents for mention of cisgender women, we reviewed the documents for issues previously identified as key to cisgender women’s use of PrEP in the broader global literature [20–22], including adherence, breastfeeding, conception, cost, condom use, pregnancy, safety, managing serodifference, sexual pleasure, side effects, and stigma.

## Results

As was expected, there is limited information addressing cisgender women’s use of PrEP in Australia. In total, 19 documents were included in the review (see Tables 1 and 2). The search generated a total of 1,380 records. 184 duplicates were removed, and 1,196 titles and abstracts were screened by a single author. At this point, 1,031 records were removed largely because they did not focus on women or were concerned with PrEP in countries outside of Australia. One hundred and sixty-five records remained and were sourced for full-text review. Thirteen records from the Google search could not be retrieved, and the remaining 152 records were independently reviewed for eligibility by two of three authors (CM & KM or HD). During this review of full-text documents, the most common reasons for records being excluded from the review were not being focused on Australia (*n* = 38), wrong population, i.e., not cisgender women (*n* = 26), not about PrEP (*n* = 32), and focused on microbicides (*n* = 16) (see Fig. 2). Fifteen documents were included at this point. The hand search of reference lists of these remaining records generated an additional 74 records of which 9 could not be retrieved, usually because of broken links to out-of-date documents. The remaining 65 records were reviewed by two authors (CM & KM) and 61 were excluded as ineligible for the review. Reasons for exclusion included wrong population i.e., not cisgender women (*n* = 26), not about PrEP (*n* = 17), not focused on Australia (*n* = 7), and outdated policies and guidelines that had been replaced by new versions already included (*n* = 8). We concluded with 19 documents that met the inclusion criteria (Fig. 2). The characteristics of the peer-reviewed articles are summarised in Table 2 and the strategy and guideline documents in Table 3.

**Table 2:** Characteristics of included peer review studies

Publication	Place of Study	Study Type	Research Aims	Population Description	Findings Specific to Women
Chan et al. (2021)	New South Wales	Quantitative	To report on the findings of the MI-EPIC study.	HIV-negative people who are Medicare ineligible, living in (or regularly visiting) NSW, and at high and ongoing risk of HIV acquisition through sexual exposure.	While the participant cohort did include women, this paper does not report on the findings specific to this population.
Chidwick et al. (2022)	National	Quantitative	1. To provide insight into the prescription of PrEP in general practice settings after its listing on the PBS. 2. To report on the sociodemographic characteristics of people who are prescribed PrEP in Australia. 3. To investigate the patterns associated with PrEP use in Australia. 4. To explore the factors associated with discontinuation and non-continuous use of PrEP.	People aged 18–74 years who had two clinical encounters during the study period at an included practice and were not diagnosed with HIV prior to 1 April 2018.	Women were more likely to have discontinued PrEP than men (OR 3.5; CI 1.1–11.7, *p* = 0.041) in univariable analysis although this was not sustained in multivariable analysis (aOR 2.7; CI 0.8–9.8, *p* = 0.113).
Dunn et al. (2022)	National	Qualitative	1. To explore the challenges PrEP presented for sexual health promotion in Australia. 2. To provide insight into how these challenges were responded to within the HIV sector.	People working in the Australian HIV health promotion sector. One participant working in the New Zealand HIV health promotion sector was also included to provide a point of comparison.	Participants noted that there has been limited consideration given to the implications of PrEP for women.
Falcão et al. (2016)	Victoria	Qualitative	To explore HIV-negative partners in heterosexual serodifferent relationships’ views about PrEP as an option for HIV prevention.	Heterosexual men and women in serodifferent relationships.	• Women were less interested in using PrEP outside conception attempts due to (1) concerns about adherence and (2) recommendations that condom use is maintained. • Women did not expect their sexual behaviour to change as a result of using PrEP.
Friedland et al. (2023)	Global	Quantitative	1. To explore which HIV and STI prevention methods women were most interested in using. 2. To investigate whether women would be interested in contraceptive Multipurpose Prevention Technologies.	Cisgender women aged 18–49 years who have had sex with a man at least once in their lifetime.	N/A article is focussed on women. Results from Australia were not disaggregated from those in United States, Europe, and Canada.
Kirby (2022)	No primary data	Opinion	In light of current strategies to end HIV transmission in Australia, this article reflects on Australia’s historic approach to the AIDS epidemic.	No primary data	Jane Costello, CEO Positive Life NSW, is quoted, stating that more support is needed for women in Australia (with regards to HIV testing, in particular).
Lane et al. (2019)	Queensland	Mixed methods	To evaluate general practitioners’ understanding of and attitudes toward PrEP for HIV prevention.	Practicing general practitioners located in Mackay, Queensland.	• Just 6.7% (*n* = 3) of participants reported being ‘very likely’ to prescribe PrEP to heterosexual people who had multiple partners. • No specific discussion of issues relating to PrEP for women.
Medland et al. (2023)	National	Quantitative	1. To understand how discontinuation impacts overall PrEP usage in Australia. 2. To ascertain how many PrEP users go on to discontinue in Australia. 3. To investigate the factors that might predict PrEP discontinuation in Australia.	Australian Government subsidised PrEP users.	More women had discontinued PrEP use than men (adjusted hazards ratio [aHR] 2.99; CI 2.65–3.38, *p* < 0.001).
Mullens et al. (2018)	Queensland	Qualitative	To describe sub-Saharan African communities in Queensland’s HIV risk status, and the potential for PrEP use in this population.	Sub-Saharan African community workers, stakeholders, and leaders.	No specific discussion of women and PrEP.
Newman et al. (2019)	National	Qualitative	To examine key stakeholder conceptualisation of disparities in access to PrEP.	Professionals working in health and social care across Australia and with HIV knowledge and experience.	Little specific discussion of women as PrEP users. Women are generally included as ‘marginalised’ populations, but no findings specific to women are presented.
Read et al. (2019)	New South Wales	Quantitative	1. To describe how people who inject drugs perceive PrEP. 2. To ascertain how willing people who inject drugs would be to begin taking PrEP, as well as their potential risk compensation.	Clients accessing services at the Kirketon Road Centre or Uniting Medically Supervised Injecting Centre.	• Of the participants who were eligible for PrEP (injecting-related risk eligibility only; *n* = 9), five were female. • Women less likely than men to have heard of PrEP (31% vs. 67%, no p-value provided)
Smith et al. (2020)	National	Qualitative	1. To understand how professionals working in the HIV sector understand the mainstreaming of PrEP prescription. 2. To explore the kinds of issues that professionals believe might arise through the mainstreaming of PrEP prescription.	Professionals working across government, university, or community-based organisations in HIV-related roles.	One participant noted that if certain GPs believe that PrEP is only for gay men, female sex workers and heterosexual women might find it difficult to access PrEP.
Smith et al. (2022)	New South Wales; Western Australia	Qualitative	To consider how clinicians conceive of PrEP users and their communities.	Clinicians in New South Wales and Western Australia who prescribe or dispense PrEP.	Women factored into clinicians ‘PrEP imaginaries’ when in serodifferent heterosexual relationships, from a high HIV-prevalence background, or working in sex work.
Vaccher et al. (2016)	New South Wales	Quantitative	To evaluate the implementation of PrEP in New South Wales healthcare settings.	HIV-negative people (mainly GBM and heterosexual women) at high-risk of acquiring HIV and taking daily oral PrEP.	Heterosexual women at high-risk of HIV acquisition are eligible to participate in PRELUDE.
Vaccher et al. (2017)	New South Wales	Quantitative	To evaluate the population validity of the PRELUDE demonstration project sample in representing high-risk GBM eligible for PrEP in New South Wales.	GBM (although the study also extended to heterosexual men and women).	Just three women were included in the original study cohort. All three women were in a serodifferent relationship and wanting to conceive naturally in the next 3 months. This cohort is excluded from all analysis in the paper.
Vujcich et al. (2023)	National	Mixed methods	1. To explore Northeast Asian, Southeast Asian, and sub-Saharan African migrants’ knowledge, attitudes, and practices regarding HIV testing and prevention. 2. To assess the barriers and opportunities surrounding HIV prevention and testing practices among Northeast Asian, Southeast Asian, and sub-Saharan African migrants.	Adults born in Northeast Asia, Southeast Asia, or sub-Saharan Africa who are now living in Australia.	• Women comprised 59.1% of the sample. • PrEP knowledge was higher among men than women (19.87% vs. 12.97% respectively, *p* = 0.001). • HIV and blood-borne virus testing rates were lower among women than men (14.05% vs. 18.37% respectively, *p* = 0.050).

**Table 3:** Characteristics of included guidelines, policies and strategies

Document	Problem To Be Addressed	Population Description	Discussion Specific to Women
ASHM National PrEP Guidelines	Update of the 2018 ASHM PrEP guidelines, designed to support clinicians in their HIV risk assessment, prescribing, and monitoring of PrEP.	All people at-risk of acquiring HIV, with a specific focus on MSM; trans and gender diverse people; heterosexual people; and people who inject drugs.	• The guidelines make specific reference to PrEP being highly effective among heterosexual men and women and note the need to strengthen current HIV prevention strategies for this population (and others). • Notes that on-demand PrEP should not be prescribed to cisgender women. • Specific section on pregnancy and HIV, noting that women’s risk of HIV increases during pregnancy. • Female sex workers are mentioned, although low rates of HIV are reported among this population. • Provides guidance for heterosexual women to commence PrEP seven days before travelling. • Notes that adherence has been a particular issue for cisgender women using PrEP.
NSW HIV Strategy 2021–2025	The virtual elimination of HIV transmission in NSW.	All people at-risk of or living with HIV. Prevention strategies are specifically targeted toward MSM, but initiative suggested for sex worker peer outreach, Aboriginal services, and Needle and Syringe programs.	This document contains little discussion of women. It does, however, point toward a need for a renewed interest in heterosexual people at-risk of HIV acquisition (including culturally and linguistically diverse people and women who are the sexual partners of MSM). Aboriginal people (gender not specified) are also included as a population requiring renewed focus. Women could be included in a number of the highlighted priority populations: as Aboriginal people; people from or who travel to countries with high HIV prevalence; people who inject drugs; sex workers and their clients; people who are in or have recently been in custodial settings; or sexual partners of members of priority populations.
Eighth National HIV Strategy 2018–2022	Guiding principles to support a high-quality, evidence-based, and equitable response to HIV in Australia.	All people at-risk of or living with HIV. Priority populations include people living with HIV; gay men and other MSM; Aboriginal and Torres Strait Islander people; culturally and linguistically diverse people from high HIV prevalence countries, people who travel to these countries, and their partners; sex workers; people who inject drugs; people in custodial settings; and trans and gender diverse people.	While cisgender women are not included as a distinct priority population, this document does acknowledge that women are included within almost all priority populations. Given this, the document emphasises the importance of implementing a gendered lens to all elements of the Australian HIV response. Specific mention is made of Aboriginal and Torres Strait Islander and CALD women’s needs; however, there is no mention of women in relation to PrEP.

**Table 4:** Issues specifically identified as significant to cidgender women

Document	Adherence	Breastfeeding	Conception	Condom use	Cost	Pregnancy	Safety	Sero-discordance	Sexual pleasure	Side effects	Stigma
ASHM (2021)	x	x	x	x	(x)	x	x	x		(x)	(x)
Chan (2022)	(x)				(x)						
Chidwick (2022)	x				(x)						
Commonwealth (2018)				(x)							(x)
Dunn (2022)				x				x			(x)
Falcão (2016)	x		x	x	x			x	(x)	x	(x)
Friedland (2023)				x	x		x			x	
Kirby (2022)											
Lane (2019)								x			
Medland (2023)	x					x					
Mullens (2018)					(x)					(x)	(x)
Newman (2019)				x	x			x			
NSW Health (2018)	(x)			(x)	(x)					(x)	(x)
Read (2019)										(x)	
Smith (2020)											(x)
Smith (2022)	(x)			(x)	(x)			x			
Vaccher (2016)	(x)	x	x	(x)		x	x			(x)	
Vaccher (2017)											
Vujcich (2023)			x	x							

x = specifically discussed regarding cisgender women’s use of PrEP; (x) = discussed, but not specifically about cisgender women

The 19 included documents incorporated 17 different studies, with two documents reporting on various initial components of the PRELUDE study [25, 26] and a further two documents reporting the same qualitative research project undertaken with clinicians and PrEP prescribers [27, 28]. All reviewed studies were published between 2016 and 2023. Peer-reviewed academic publications included in the review drew upon a range of research designs, including qualitative methods (*n* = 7) [27–32]; cross-sectional surveys (*n* = 3) [25, 33, 34]; opinion and text (*n* = 1) [35]; analysis of secondary data (*n* = 1) [36]; mixed method studies (*n* = 2) [37, 38]; single-arm trials (*n* = 1) [39] longitudinal observational studies (*n* = 1) [40]; and a protocol paper (*n* = 1) [26]. Six studies were conducted at a national scale [29, 30, 33, 36, 37, 40]; four were conducted in New South Wales [25, 26, 34, 39]; two in Queensland [32, 38]; two across Western Australia and NSW in the same study [27, 28]; and one in Victoria [31].

Studies were particularly focused on populations deemed to be at-risk of HIV acquisition (*n* = 8), including migrant communities from HIV endemic countries [32, 37], people who use injecting drugs [34], and broad GBM and heterosexual populations [25, 26, 31, 33, 39]. A further group of studies focused on healthcare professionals in the HIV sector to establish their attitudes and experiences regarding PrEP prescribing (*n* = 5). Three studies interviewed clinicians and GPs [27, 28, 38], and a further two interviewed HIV professionals [30] or health promotion staff [29], many at a time when PrEP was not yet available through the PBS. A final, smaller number of studies were concerned with secondary analysis of PrEP prescribing data [36, 40]. Two strategy documents (one national and one specific to New South Wales) [1, 41] and one national guideline [42] were also included in the review. Each of these documents target a broad population of people considered at-risk of HIV acquisition but include at least some mention of PrEP use specifically for cisgender women.

When examining the extent to which Australian-focused documents addressed issues that have been raised as significant for female PrEP-users, few specifically discussed these factors in relation to how they might impact cisgender women differently to men (see Table 1). The ASHM prescribing guidelines most thoroughly address these issues from cisgender women’s perspectives, with specific mention of adherence, breastfeeding, conception, pregnancy, safety, and serodifference [42]. Other sources that particularly discuss these issues with regard to their specific impacts for cisgender women include Friedland [33], with discussion of condom use, cost and side effects, and Vaccher [26], which included breastfeeding, conception, pregnancy, and safety.

Across the eleven issues specifically identified as being significant for cisgender women, PrEP use in a context of serodifference was most frequently discussed [28–31, 38, 42], often in relation to a desire to conceive children [31, 42]. Condom use was also frequently mentioned with regard to cisgender women [29–31, 33, 37, 42], as was conception [26, 31, 37, 42] and adherence [31, 36, 40, 42]. The remaining identified issues were rarely discussed with regard to cisgender women, with sexual pleasure specifically not mentioned in any discussion of their potential use of PrEP. Falcão et al.’s [31] discussion of PrEP use in heterosexual couples with differing HIV status suggests that pleasure is a male concern associated with reducing condom use and not significant for cisgender women when choosing to use PrEP in the context of conception.

## Discussion

The success of PrEP among GBM in Australia suggests potential for the elimination of HIV transmission in Australia, but this will require thinking about access in other populations. While PrEP is theoretically available to all who are suitable or request it, little has been done to ensure that cisgender women’s access to PrEP has been specifically enabled. In this scoping review we identified 16 publications and three guideline or strategy documents that mentioned PrEP for cisgender women in any depth between 2016 and May 2023.

### Limited Focus on Cisgender Women and PrEP

Despite the focus of this review, cisgender women were often given only passing mention in peer-reviewed academic publications and, although reported on in the sample, were frequently excluded from further analysis and discussion of results. For example, Vaccher et al. [26] specifically included heterosexual cisgender women as potential participants in the PRELUDE study protocol, but there is limited discussion of cisgender women in further publications from the demonstration study. Baseline date from PRELUDE [25] is limited to three cisgender women and no elaboration on the interrelationship between issues such as breastfeeding and pregnancy with PrEP could be made due to a lack of sufficient numbers for sub-analysis. Further publications from this study also do not explicitly discuss outcomes for cisgender women for the same reason. Similarly, Chan et al. [39] examine providing free PrEP coverage to Medicare-ineligible people living in Australia in the MI-EPIC trial. Again, cisgender women were eligible to participate, but only a single woman was enrolled in the study and no further discussion focused on cisgender women’s experiences of taking PrEP was therefore analytically possible.

### Cisgender Women Rarely Specifically Identified in Existing Australian PrEP Literature

In the guideline and strategy documents, cisgender women were usually subsumed into other priority populations, such as people who use injecting drugs, Aboriginal and Torres Strait Islander or discussed in terms of sexual orientation and not sexual behaviour [2, 41, 42]. This carried through into published papers where, for example, Smith et al.’s [28] discussions of imagined PrEP users explicitly included cisgender women, but there was no discussion of their potential HIV risk outside of being part of a serodifferent heterosexual couple. Thus, cisgender women who do not identify as heterosexual are excluded from much of the discussion about PrEP, despite recent Australian evidence suggesting that some lesbian, bisexual, and queer women embedded in the broader queer community report having sexual partners who are GBM and could therefore be at more risk of HIV transmission than most heterosexual women [43]. This echoes arguments made in feminist writings about HIV and women that note how women are vulnerable to exclusion from discourse about HIV prevention other than when their inclusion is linked to a male partner – in this case a known HIV-positive partner with whom they wish to have children or who is not able to successfully manage their HIV viral suppression [44–46].

### No Specific Strategies to Reach Cisgender Women

Although several sources discussed concerns about the potential to overlook PrEP need among populations other than GBM [29, 35] and that cisgender women are less likely than men to know about PrEP [37], there was no discussion of the need to develop specific strategies or approaches that might particularly address PrEP access for cisgender women identified in this review. Australia is acknowledged as having one of the most comprehensive HIV responses in the world, premised on a strong partnership between community, government, and the healthcare sector [47]. Among GBM, advances in HIV prevention have been successful in an engaged community with a largely common identity. Policy for prescribing PrEP has therefore been premised on ‘knowledgeable patients’ able to request PrEP for HIV prevention from their healthcare provider [27].

This review of the literature supports the position that Australia is not yet in a place to consider how the success of PrEP prescribing might be extended to cisgender women in the context of eliminating HIV transmission. Our own research has indicated that healthcare providers are uncertain of PrEP prescribing for cisgender women, require cisgender women to meet higher thresholds of eligibility and acknowledge that efforts have not been made to ensure that cisgender women have access to PrEP information prior to approaching their healthcare provider [48]. Dunn, Barnett, and McKay [29] echo this point when they note that people who are not GBM, and who may be at risk of HIV, may not know about PrEP as they are not embedded in communities in which PrEP information has been made available. Among health promotion providers in their study “these groups [non-GBM] were viewed as having specific needs and concerns that needed to be addressed by the health promotion community” (p. 6). Similarly, Friedland et al.’s [33] survey of multipurpose prevention technology among 737 cisgender women (including from Australia) found that none reported using PrEP, despite the majority being interested in HIV prevention.

The models of PrEP prescribing that we currently have for cisgender women have been inherited from working with GBM populations and do not sufficiently engage with where cisgender women are in terms of their access to information about PrEP and their confidence to request PrEP from providers. Indeed, current approaches to HIV prevention for cisgender women in Australia premised on an informed patient requesting PrEP run counter to what we know are their experiences of health contexts that are not particularly skilled at taking sexual histories or exploring HIV risk outside of GBM populations [49–51]. International research suggests that non-GBM patients gain access to HIV prevention only when providers initiate conversations about PrEP [52, 53] and that PrEP messaging should be normalised as an available option for cisgender women [54].

### Women’s Specific Needs Not Acknowledged

We specifically examined issues that have been identified as significant for cisgender women in the international literature. There are suggestions from the included studies that Australian cisgender women face specific challenges in PrEP adherence. Both Chidwick et al. [40] and Medland et al. [36] note that cisgender women were more likely to discontinue PrEP use than men, and although they speculate that this may be appropriate in the context of attempts to conceive, the data suggest that we need to know more about cisgender women’s specific engagement with PrEP. International literature has noted similar low adherence and high discontinuation among cisgender women [20]. Taken with research showing that PrEP becomes ineffective in cisgender women after fewer missed doses than in men [55], we need specific strategies to address adherence in cisgender women. PrEP prescribing guidelines specifically speak to the need to educate cisgender women for better adherence, but such strategies remain largely undeveloped [42].

While a handful of the included references discussed pregnancy, breastfeeding and conception, these specifically female PrEP concerns have not received much attention in Australia. Although the safety of PrEP during these life stages has been proven, specific guidelines are needed to ensure both provider and patient knowledge. The anticipated surge in PrEP use among cisgender women during pregnancy and breastfeeding since listing on the PBS has not yet eventuated [55], but healthcare providers must prepare for a potential increase in demand.

Current or intended condomless sex is indicated for prescribing PrEP to heterosexual patients in the ASHM guidelines [42], and several other studies included in the review discussed how experience or attitudes to condoms interact with cisgender women’s potential PrEP use. Women interviewed by Falcão et al. [31] were cautious about PrEP and preferred to continue using condoms unless actively trying to conceive. This was not matched by similar attitudes among male participants, highlighting the potential for conflict in serodifferent relationships. Health promotion practitioners welcomed the opportunity provided by PrEP to move away from promoting condoms among serodifferent couples [29], however other healthcare providers were particularly concerned about cisgender women’s ability to insist on condom use and that equitable access to PrEP should, but does not presently, account for this [30].

### Strengths and Limitations

There are several limitations to this scoping review. The purpose of the review was not to assess the quality of these documents. The aim, rather, was to understand what is currently known about this topic in Australia and all documents were therefore included regardless of quality, in keeping with scoping review methodology [23]. One limitation is that research could have been overlooked due to the search term and databases selected. While the researchers tried to account for this limitation through reference list searches of identified documents, there appear to be several apparent omissions. For example, the review did not pick up any strategy documents for Australian states and territories other than NSW: we specifically verified that other strategy documents did not meet the inclusion criteria. We also note that the search did not identify Health Equity Matters’ (formerly AFAO) consensus document Agenda 2025, which specifically documents the steps needed to eliminate HIV by 2050 and makes specific reference to women [1].

### Conclusion and Recommendations

While there is opportunity in current guidelines to prescribe PrEP to cisgender women in Australia, little is being done to specifically consider their access to and use of PrEP. There is currently limited recognition that cisgender women may need alternative approaches to accessing information and prescribing, and that healthcare providers’ (particularly GPs) knowledge of and comfort with prescribing PrEP to cisgender women is limited [48]. Current training for GPs and demarcation of sexual health clinics as spaces for GBM, not cisgender women [48], are not effective if we are to enhance women’s access to PrEP.

As in the US [55], we need to think specifically about how to make PrEP accessible for cisgender women. We recommend that standard sexual health screens for cisgender women are broadened to include HIV testing, and that efforts are made to better equip health care providers with both the time and inclination to facilitate discussion of HIV risk with cisgender women. Enhanced sexual health services at additional locations, such a travel clinics may also be useful for addressing cisgender women’s HIV risk [16, 56]. Additionally, if prescribing guidelines continue to be premised on the ‘informed PrEP user’ [28], educational materials must be expanded to be perceived as relevant to cisgender women and made available in locations where they can be accessed by this population.

There has been specific emphasis on enabling PrEP access for non-Medicare eligible GBM [57] and our understanding of which cisgender women may be vulnerable to HIV acquisition suggests that similar efforts are needed to reach cisgender women from multicultural backgrounds and with international connections. The MI-EPIC trial showed that PrEP can be delivered to Medicare-ineligible patients [39], but few cisgender women were enrolled in this trial and delivery of PrEP was reliant on publicly funded sexual health clinics. We do not know how Medicare-ineligible cisgender women might access PrEP online. Barriers to knowledge about and access to PrEP are likely to be more pronounced among multicultural cisgender women and will require specific culturally competent approaches [58].

Similarly, non-heterosexual women should not be excluded from discussions of and access to information on PrEP, as their risk of HIV acquisition may not be as negligible as previously assumed. This demands a change in the language of current guidelines such that route of transmission (heterosexual) is not conflated to represent women’s sexual identities, thereby excluding women who may identify as queer, but engage in unprotected sex with both heterosexual and/or bisexual men. As noted by Tony Kirby [35], “until Australia effectively targets all people at risk of HIV with nuanced treatment and prevention efforts, the elimination of HIV transmission will not be achieved”(p. e461). Making sure that we assure cisgender women’s access to PrEP is part of this elimination process.
